# Case Report: Pemphigus in Young Patients With Thymic Anomalies

**DOI:** 10.3389/fmed.2022.844223

**Published:** 2022-02-24

**Authors:** Naiyu Lin, Xiaoli Li, Yuejiao Lang, Jiande Han

**Affiliations:** ^1^Department of Dermatology, The First Affiliated Hospital, Sun Yat-sen University, Guangzhou, China; ^2^Department of Pathology, The First Affiliated Hospital, Sun Yat-sen University, Guangzhou, China

**Keywords:** pemphigus, thymus, autoimmune disease, young, T cell

## Abstract

Pemphigus is an autoimmune disease that occurs mainly in elderly individuals. Young patients with pemphigus are rare, and the risk factors are unclear. The thymus is associated with a variety of autoimmune diseases, and there have been reports of pemphigus complicated with thymic diseases. Here, we report three cases of young patients with pemphigus that were associated with thymic anomalies. We suggest that thymic anomalies may be a risk factor for the early onset of pemphigus and may be associated with increased severity of the disease. Interventions for thymic diseases have certain benefits for improving the effect of treatments and prognosis of these patients.

## Introduction

Pemphigus is a life-threatening autoimmune bullous disease that involves the skin and mucous membranes. It commonly affects elderly people, with distribution across all races (1). The thymus is one of the most important immune organs that ensure T cell tolerance. Thymus-related diseases are associated with many autoimmune diseases including pemphigus (2). Currently, factors that may contribute to an earlier onset of pemphigus (at a younger age) and/or exacerbate the severity of the disease are unknown. In this article, we report three cases of pemphigus with thymic changes. None had a history, symptoms of other autoimmune diseases, nor a family history of any related autoimmune disease or thymic disease.

## Case 1

**Figure 1:**
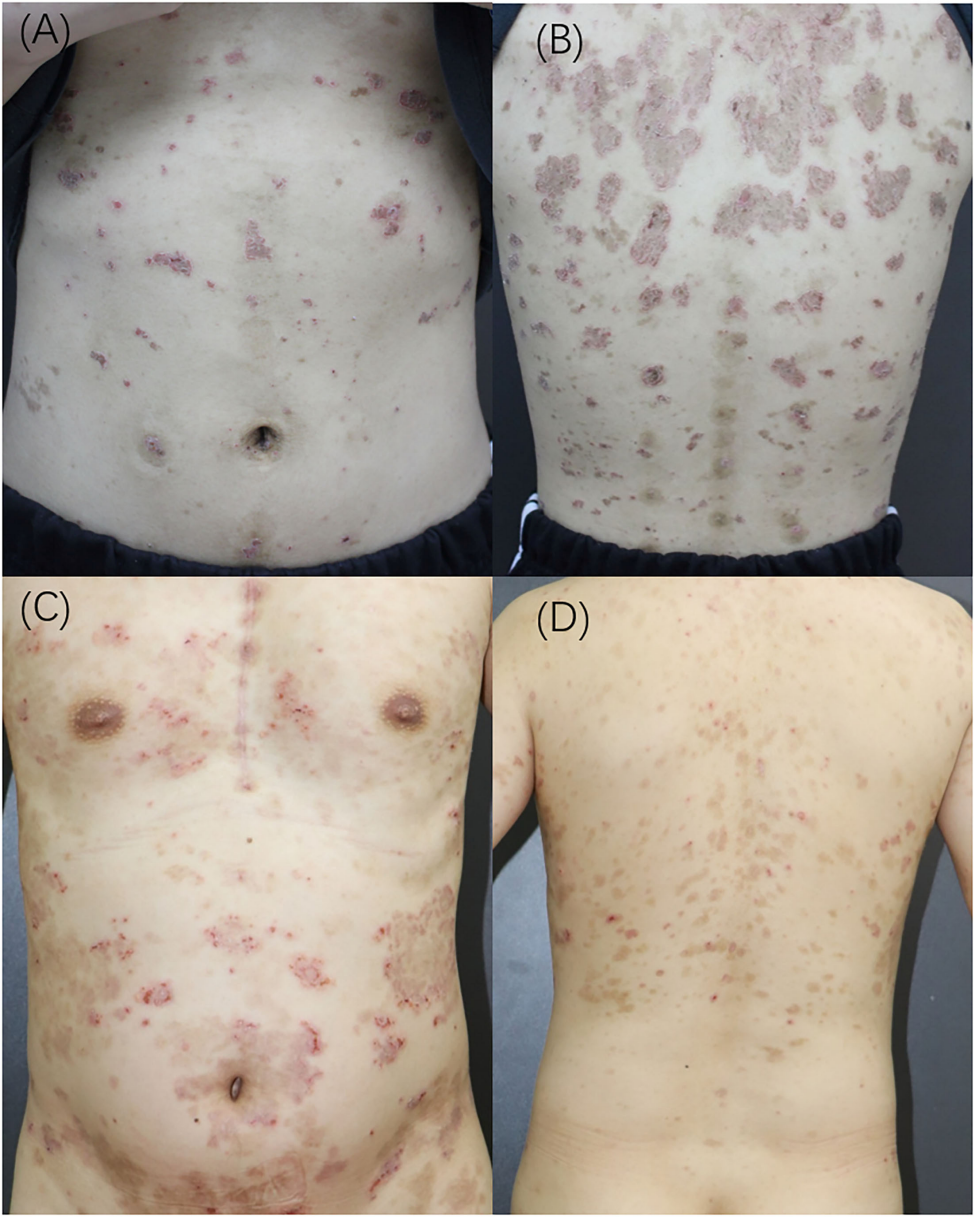
**(A,B)** Clinical presentations of Case 1; **(C,D)** Clinical presentations of Case 2.

**Figure 2:**
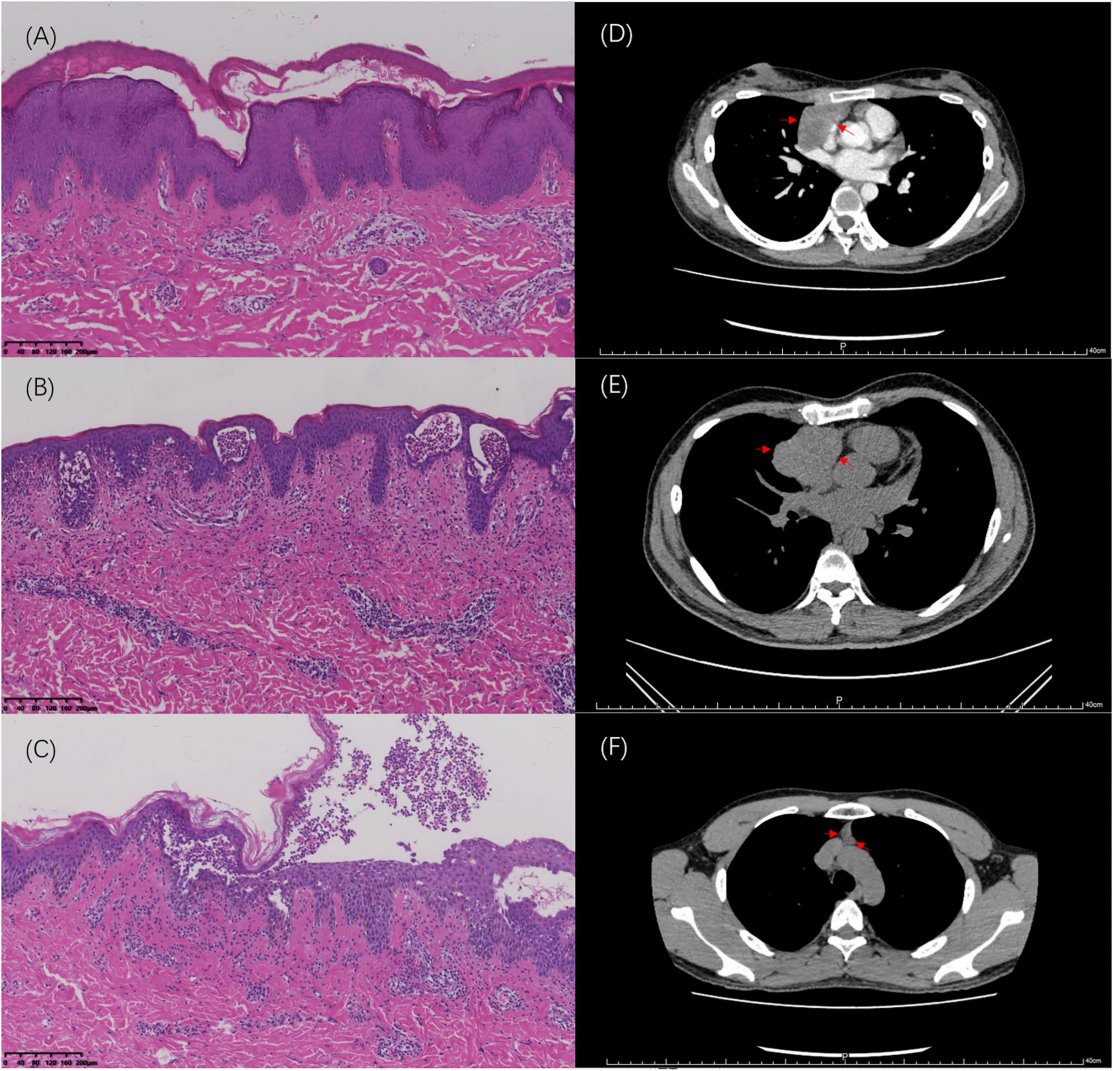
Hematoxylin and eosin (HE) stained sections of skin lesions (100×) and imaging changes of the thymus. **(A)** Case 1: Formation of substratum corneum fissures is observed. **(B)** Case 2: Blisters in the epidermis are filled with neutrophils. **(C)** Case 3: Bullous formation in the epidermis with acantholysis cells is visible. **(D)** Thymic imaging via CT scan for Case 1 at first hospitalization. **(E)** Thymic imaging via CT scan for Cases 2 before surgery. **(F)** Thymic imaging via CT scan for Case 3 at 2 years after his first admission.

An 18-year-old woman presented with a 4-month history of widespread erythema, scaling, and blisters (Figures 1A,B). Physical examination revealed a positive Nikolsky's sign, and laboratory examination revealed that she was desmoglein 1 (Dsg-1) antibody-positive and desmoglein 3 (Dsg-3) antibody negative. Blood counts were assessed upon admission, and the total lymphocyte count and lymphocyte percentage remained normal. Histology revealed the formation of substratum corneum fissures (Figure 2A). Immunofluorescence indicated IgG and C3 deposition at the same site, which was consistent with a diagnosis of pemphigus foliaceus (PF). Routine computed tomography (CT) on the day of admission incidentally revealed a mediastinal mass (Figure 2D), which was likely to be a thymoma, without obvious malignant features. The patient was administered with prednisone; however, it was resistant to the initial treatment regimen. Approximately 1.5 mg/kg/day of prednisone was administered for more than 1 month before the rash improved. The patient did not initially undergo surgical treatment for the thymoma as a needle biopsy of the thymus revealed benign features. However, she eventually underwent surgery to remove the thymic mass due to repeated episodes of pemphigus. As the needle biopsy revealed that the mass was benign, the examination of the thymus confirmed the diagnosis. Moreover, the patient's rash improved rapidly after the operation, and only 4 mg of methylprednisolone per day was needed as maintenance therapy for 3 months (Table 1).

**Table 1:** Clinical characteristics of included cases.

**Case**	**Age**	**Sex**	**Diagnoses**	**Autoantibodies deteced**	**Thymic changes observed**				**Therapy**	**Outcomes**
					**Time**	**Size (mm)**	**Character**	**Operation**		
Case1	18	Female	PF	Dsg1(+)Dsg3(–)	At time of diagnosis	55 × 42 × 10	Benign	Yes	Prednisone	Responded well after surgery
Case 2	35	Male	PH	Dsg1(–)Dsg3(–)	Prior to diagnosis	57 × 22 × 10	Borderline	Yes	Prednisone	Responded well
Case 3	22	Male	PV	Dsg1(–)Dsg3(+)	After diagnosis	52 × 43 × 25	Benign	None	Prednisone + CTX	Treatment-resistant

*Dsg, desmoglein; PF, pemphigus foliaceus; PH, pemphigus herpetiformis; PV, pemphigus vulgaris; CTX, cyclophosphamide*.

## Case 2

A 35-year-old man was admitted to the hospital with generalized small pruritic blisters that began 7 months prior (Figures 1C,D). These lesions were diagnosed as eczema at his first outpatient visit; however, the corresponding treatment was not effective. Ten months prior to the onset of his symptoms, he underwent surgery for a thymic tumor, which was discovered during an annual physical examination (Figure 2E). Prior to thymus surgery, blood counts was not suggestive of abnormal lymphocyte counts and proportions. The tumor was diagnosed as a type AB thymoma based on the WHO classification method. Further physical examination revealed a negative Nikolsky's sign, and serum tests were negative for antibodies to Dsg and bullous pemphigoid. Pathology results described the formation of blisters in the epidermis as well as a large number of neutrophils in the blisters (Figure 2B). Lymphocytes, neutrophils, and eosinophils had also infiltrated the superficial dermis. Immunofluorescent revealed the intercellular staining of IgG (++), IgA (+), and C3 (±) among epidermal spinous cells. Based on the above findings, we made a diagnosis of pemphigus herpetiformis (PH). The patient responded well to treatment. The initial use of 1 mg/kg/d of prednisone controlled his symptoms, and dose was tapered successfully without recurrence (Table 1).

## Case 3

The third patient was a 22-year-old man whose chief complaint was repeated blisters and oral ulcers for 2 months. He had all the typical manifestations of pemphigus vulgaris (PV), including typical pathological (Figure 2C) and immunofluorescent manifestations (i.e., intercellular staining of IgG (+), C3 (±), and C1 (±) among epidermal spinous cells), high titer Dsg-3 antibodies, and positive Nikolsky's sign. The patient developed treatment resistance during the initial therapy, and no improvement was observed in spite of administering prednisone at a dose of 1.5 mg/kg/d for 2 weeks. Therefore, we increased the dose of prednisone to 2 mg/kg/d and added a dose of cyclophosphamide 0.4 g/time, administered over 2 consecutive days, once a month. During the next 8 months follow-up period, his prednisone dose could hardly be tapered, and he also experienced several recurrent episodes. Surprisingly, his chest CT showed normal findings during his first admission, but thymic hyperplasia (measuring ~22 × 10 × 51 mm) was found after 2 years of follow-up (Figure 2F). However, the thymic anomaly had no effect on the peripheral lymphocyte count and proportion. The mass was closely observed and it has shown a tendency to shrink over the last 21 months of follow-up. It showed no signs of malignancy, and thoracic surgeons insisted on close follow-up observation rather than surgery (Table 1).

## Discussion

According to recent studies, among inpatients with pemphigus, simultaneous autoimmune disorders were inversely associated with increasing age (3). Thymic tumors were also associated with a variety of autoimmune diseases, including pemphigus. However, pemphigus was primarily an autoantibody-driven disease; hence, its link to thymic dysfunction was intriguing. In recent years, it has been found that auto-reactive CD4+ T cells were essential for the induction and perpetuation of pemphigus through the interaction with B cells to produce autoantibodies (4). There are various T cell subtypes: Th2 cells and Tfh cells support self-reactive B cell survival, differentiation, and subsequent antibody production; Th17 cells promote inflammation response; and Treg cells prevent autoimmunity (5, 6). It has been proven that the transcription factor Aire contribute to the process of thymus-induced central tolerance by promoting Dsg 3 expression in the medullary thymic epithelial cells, which serve as self-antigens in negative selection (7). Therefore, the three cases in this study may be erupted by the disturbance of central tolerance and induction of auto-reactive T cells. Furthermore, those elderly patients with pemphigus but without thymic disease may be more likely to experience decreased Tregs numbers and function.

From these cases, it was suggested that the age of onset and disease severity of pemphigus seems to be related to the presence of thymic changes. Additionally, thymic tumors can occur before, after, or simultaneously with pemphigus. We also found that the removal of thymic tumors may help improve the symptoms of pemphigus. PNP may be considered initially when confronted by a patient with pemphigus that was complicated with a tumor. However, none of the three patients had characteristics of PNP, such as severe mucosal involvement, polymorphic lesions, or positive serological results for multiple autoantibodies (8). Furthermore, we also noticed that besides PNP, thymic changes could complicate many types of pemphigus such as PV, PF, and PH. PH is a rare type of pemphigus, which presents with pruritic rash and needle tip to bean-size blisters with negative Nikolsky's sign. PH could transform to either PV, PF, or pemphigus erythematosus (PE). Its relatively good prognosis may have partially contributed to the smooth treatment course of the second patient; however, the fact that the thymic tumor had been removed before the onset of pemphigus cannot be ignored. In the third patient, the increase in the size of the thymus was unpredictable, and this may be involved in the early development of PV. However, the changes may have been too subtle to be identified through imaging upon admission to the hospital. Therefore, it was necessary to perform chest CT regularly in such patients.

Based on these clinical findings, we emphasized that the causes of pemphigus in young patients should be further explored. This will greatly help in reducing the use of medication and shorten the course of treatment. Thymic changes, whether benign or malignant, could occur in any form of pemphigus, and this may be one of the most important risk factors for the development of pemphigus in young individuals. Hence, close follow-up was necessitated, and removal of the related thymic tumor should be considered if necessary (9).

## Data Availability Statement

The original contributions presented in the study are included in the article/Supplementary Material, further inquiries can be directed to the corresponding authors.

## Ethics Statement

The studies involving human participants were reviewed and approved by IEC for Clinical Research and Animal Trials of the First Affiliated Hospital of Sun Yat-sen University. The patients/participants provided their written informed consent to participate in this study.

## Author Contributions

NL finalized the patients, wrote the manuscript, prepared figures, and approved the final draft. XL participated in the clinical diagnosis and treatment of the patients and approved the final draft. YL participated in the pathological diagnosis, prepared figures, and approved the final draft. JH proposed the concept that we should pay attention to the possibility of thymic changes in young pemphigus patients, reviewed the manuscript, and approved the final draft. All authors contributed to the article and approved the submitted version.

## Conflict of Interest

The authors declare that the research was conducted in the absence of any commercial or financial relationships that could be construed as a potential conflict of interest.

## Publisher's Note

All claims expressed in this article are solely those of the authors and do not necessarily represent those of their affiliated organizations, or those of the publisher, the editors and the reviewers. Any product that may be evaluated in this article, or claim that may be made by its manufacturer, is not guaranteed or endorsed by the publisher.
