# Supplementary figures and images for: Case Report: Pemphigus in Young Patients With Thymic Anomalies
Source: Front Med (Lausanne). 2022 Feb 24;9:844223. doi: 10.3389/fmed.2022.844223 (PMC8907624; doi:10.3389/fmed.2022.844223)

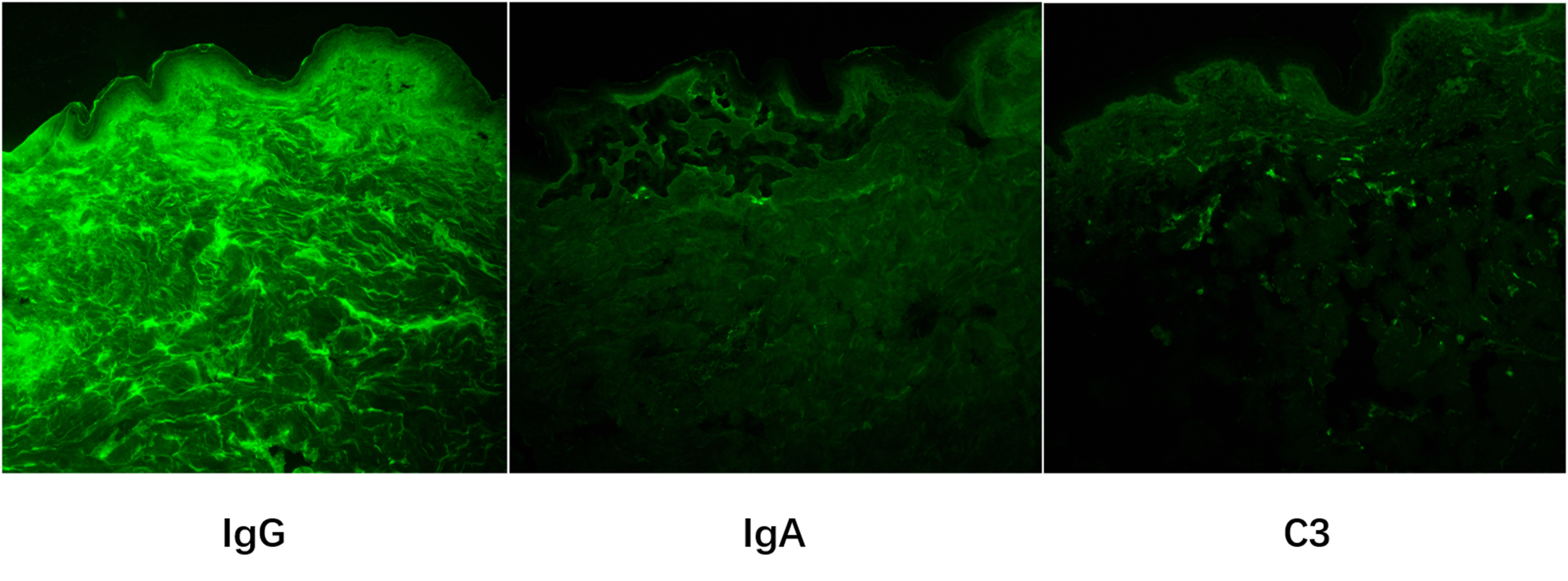

Supplement: Supplementary file 1 [file Image_1.TIF]

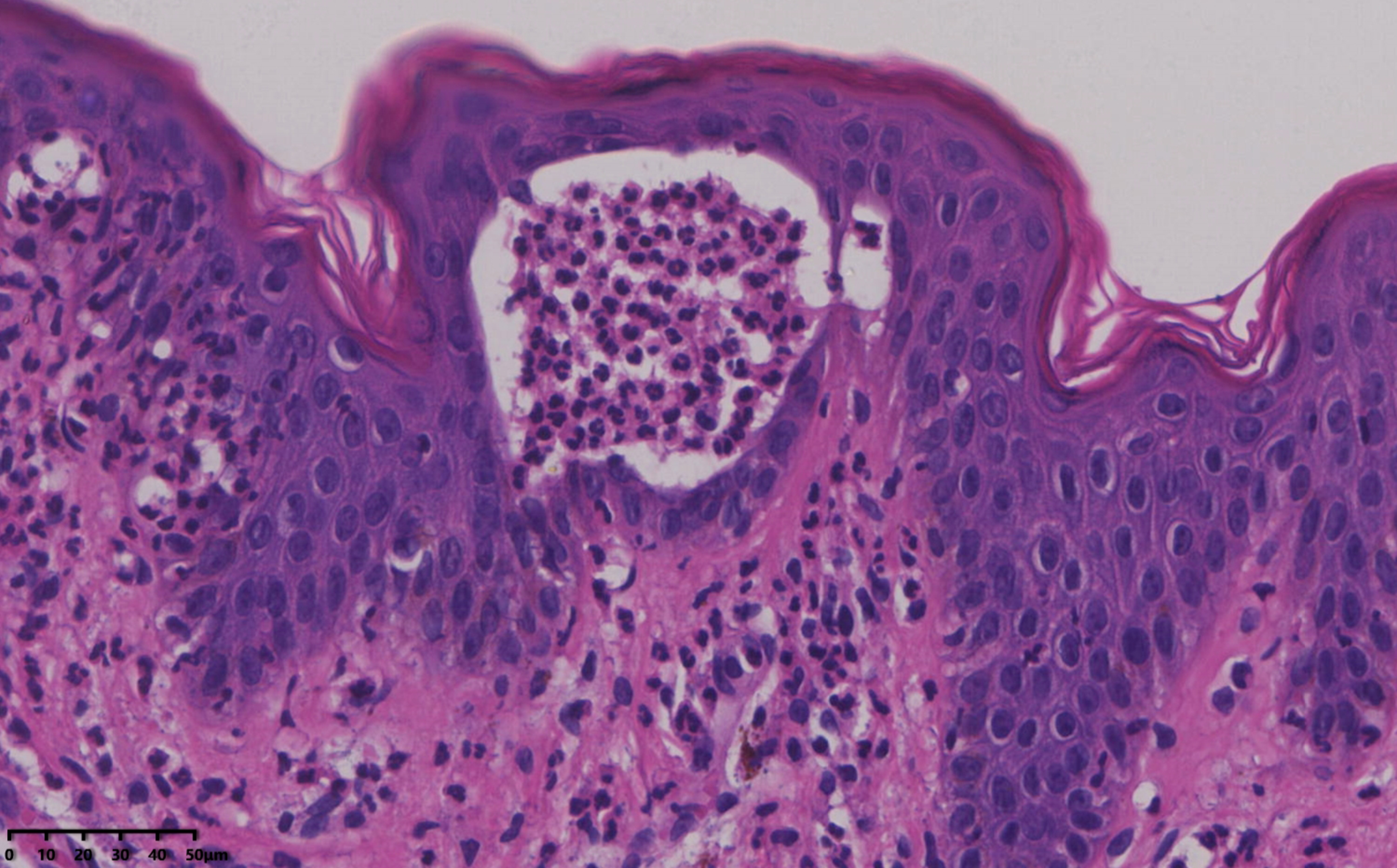

Supplement: Supplementary file 2 [file Image_2.JPEG]

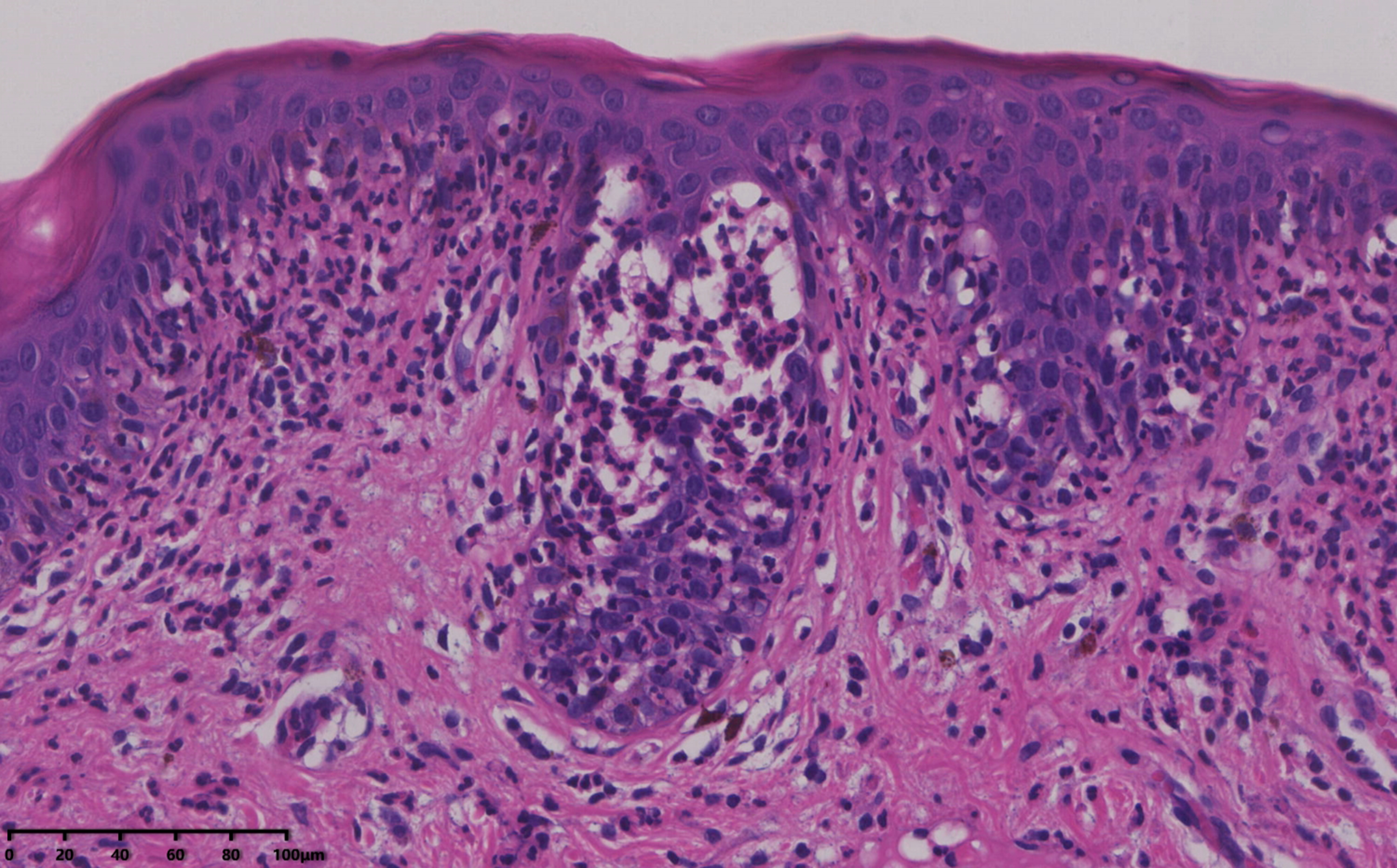

Supplement: Supplementary file 3 [file Image_3.JPEG]
